# A putatively new family of alphaproteobacterial chloromethane degraders from a deciduous forest soil revealed by stable isotope probing and metagenomics

**DOI:** 10.1186/s40793-022-00416-2

**Published:** 2022-05-08

**Authors:** Eileen Kröber, Saranya Kanukollu, Sonja Wende, Françoise Bringel, Steffen Kolb

**Affiliations:** 1 Max-Planck Institute for Marine Microbiology, Celsiusstrasse 1, 28359 Bremen, Germany; 2 Microbial Biogeochemistry, RA Landscape Functioning, ZALF Leibniz Centre for Agricultural Landscape Research, Müncheberg, Germany; 3 Génétique Moléculaire, Génomique, Microbiologie (GMGM), Université de Strasbourg, UMR 7156 CNRS, Strasbourg, France; 4 Thaer Institute, Faculty of Life Sciences, Humboldt University of Berlin, Berlin, Germany

**Keywords:** Stable isotope probing, Chloromethane, Volatile organic chemicals, Methylotrophy

## Abstract

**Background:**

Chloromethane (CH_3_Cl) is the most abundant halogenated organic compound in the atmosphere and substantially responsible for the destruction of the stratospheric ozone layer. Since anthropogenic CH_3_Cl sources have become negligible with the application of the Montreal Protocol (1987), natural sources, such as vegetation and soils, have increased proportionally in the global budget. CH_3_Cl-degrading methylotrophs occurring in soils might be an important and overlooked sink.

**Results and conclusions:**

The objective of our study was to link the biotic CH_3_Cl sink with the identity of active microorganisms and their biochemical pathways for CH_3_Cl degradation in a deciduous forest soil. When tested in laboratory microcosms, biological CH_3_Cl consumption occurred in leaf litter, senescent leaves, and organic and mineral soil horizons. Highest consumption rates, around 2 mmol CH_3_Cl g^−1^ dry weight h^−1^, were measured in organic soil and senescent leaves, suggesting that top soil layers are active (micro-)biological CH_3_Cl degradation compartments of forest ecosystems. The DNA of these [^13^C]-CH_3_Cl-degrading microbial communities was labelled using stable isotope probing (SIP), and the corresponding taxa and their metabolic pathways studied using high-throughput metagenomics sequencing analysis. [^13^C]-labelled Metagenome-Assembled Genome closely related to the family *Beijerinckiaceae* may represent a new methylotroph family of Alphaproteobacteria*,* which is found in metagenome databases of forest soils samples worldwide. Gene markers of the only known pathway for aerobic CH_3_Cl degradation, via the methyltransferase system encoded by the CH_3_Cl utilisation genes (*cmu*), were undetected in the DNA-SIP metagenome data, suggesting that biological CH_3_Cl sink in this deciduous forest soil operates by a *cmu*-independent metabolism.

**Supplementary Information:**

The online version contains supplementary material available at 10.1186/s40793-022-00416-2.

## Background

Chloromethane (CH_3_Cl) is a well-documented halocarbon and one of the most abundant volatile organic compounds in the atmosphere. The global atmospheric concentration of CH_3_Cl is currently 600 parts per trillion (ppt) and is responsible for 17% of halogen-dependent ozone depletion in the stratosphere [1, 2]. Abiotic and biotic processes in plants, grassland, soil, salt marshes, and oceans are the key drivers of global chloromethane formation [2–6]. The major CH_3_Cl sinks in the troposphere are photochemical processes involving OH radicals [1, 7], i.e. currently estimated as 2.8 Tg year^−1^ [2]. Furthermore, microbial degradation in top soils is a major global sink of the atmospheric trace compound CH_3_Cl [8–10]. However, discrepancies in the CH_3_Cl flux estimates (range between 1.5 and 2.5 Tg year^−1^) have been observed between known sources and calculated sinks [1]. Thus, the imbalance of the global budget of CH_3_Cl is due to underestimated sinks, notably forest soils and their microbial degradation activity.

Biological sink activity within the forest soils may represent a significant CH_3_Cl contribution of as much as 1.0 Tg year^−1^ [2, 7]. Strong evidence of the CH_3_Cl metabolism by soil bacteria, by both oxidation and hydrolysis in earlier investigations suggests that they are the potential sinks of CH_3_Cl [11–13]. Keppler et al. [14] suggested that microbial soil sink could be 1600 Gg year^−1^, which might still be considerably underestimated due to the ubiquitous presence of yet unquantified impact of CH_3_Cl degraders in soils [13].

The microorganisms active in terrestrial sink of CH_3_Cl are poorly characterized compared to other atmospheric trace gases such as methane or nitrous oxide. Among methylotrophs, a few are capable of utilizing CH_3_Cl as their primary growth substrate [15–17]. CH_3_Cl-degrading bacteria were isolated from diverse natural environments [11, 13, 18], including representatives from Alpha-, Beta-, and Gammaproteobacteria, Actinobacteria, and Firmicutes [15, 19]. Taxonomically diverse CH_3_Cl-degrading bacteria have been isolated from the soil such as alphaproteobacterial genera *Aminobacter*, *Hyphomicrobium*, *Methylorubum*, *Methylocella*, *Acidicaldus* and *Bradyrhizobium* [13, 20, 21]. The most comprehensively characterized metabolic pathway of aerobic CH_3_Cl degradation is the *cmu* pathway, studied in detail in a model methylotroph, *Methylorubrum extroquens* (previously *Methylobacterium extorquens*) strain CM4 [22]. Genes *cmuA* and *cmuB* encoding methyltransferases essential for CH_3_Cl dehalogenation have been detected in various environments, including forest soils [16, 20, 23–25]. However, the absence of *cmu* genes in active CH_3_Cl degraders in metagenomics and labelling studies suggests this pathway is not the only CH_3_Cl degradation pathway [15, 17, 19] and limits CH_3_Cl-degrading bacteria detection by overlooking *cmu*-independent microbial sink in soils.

Stable isotope probing (SIP) is a powerful method used in microbial ecology in order to identify specific functional groups of microorganisms that incorporate distinct (labeled) substrates without knowing how to cultivate them in standard laboratory conditions. When combined with metagenomics, SIP allows scientists to gain an unparalleled insight into in situ activities of low-abundance and/or novel taxa [26], since only microorganisms involved in the cycling of the labeled substrates are sequenced.

Such a SIP approach enabled the identification of key bacterial degraders of naturally occurring CH_3_Cl in a temperate German European beech forest soil [23] using 16S rRNA gene-targeted sequencing. The major consumers of CH_3_Cl-derived carbon belonged to the family *Beijerinckiaceae* of the class of Alphaproteobacteria [23]. However, in these methylotrophic aerobes, the metabolic CH_**3**_Cl-utilization pathway has not been identified. Also, the precise phylogenetic affiliation of these Alphaproteobacteria CH_3_Cl degraders remained elusive. Using the same forest soil location where these consumers of CH_3_Cl-derived carbon have been identified, this study aims to identify genomic and metabolic traits of active aerobic microbial CH_3_Cl degraders [23]. Here, a combination of DNA-SIP and high-throughput metagenomic sequencing was used to unravel the active CH_3_Cl-degrading bacteria and to study their methylotrophic metabolism.

## Methods

### Environmental sampling and microcosm incubations

Soil samples and degraded leaf samples were taken from the Steigerwald, Germany, in June 2017. Degraded leaf samples were samples above the mineral soil and consisted of rotten and partly decomposed leaf material. Three organic soil samples, three mineral soil samples and three degraded leaf samples were extracted, transported to the laboratory on the same day, and stored overnight at 4 °C before processing in the following morning. DNA stable isotope labelling (SIP) experiments were set up with three biological replicates to determine the most active layers for chloromethane degradation. Furthermore, no substrate-added control incubations and control incubations with potassium cyanide (20 mM) were set up to determine intrinsic chloromethane formation and non-microbial chloromethane dissipation, respectively.

Two grams of organic or mineral soil or degraded leaves were incubated in a 125-mL serum vial and flushed with synthetic air (Linde AG, Germany). Microcosms were set up in three biological replicates by adding [^13^C]-CH_3_Cl (Campro Scientific, Germany) or [^12^C]-CH_3_Cl (Linde AG, Germany), to a final concentration of 250 mmol^−1^ g^−1^ DW (dry weight). In more detail, serum vials were sealed with butyl rubber stoppers, evacuated to a level of 0.01 bar and flushed three times with synthetic air containing 20% oxygen and 80% dinitrogen gas (Linde AG, Germany) to a pressure of 1.2 bar. Finally, CH_3_Cl (Linde, Germany) was added using a 100 μL gas-tight syringe (Hamilton, Romania). Gas samples were taken with gas-tight syringes and stored in 3-mL, pre-evacuated Exetainers (Labco Limited, England) for further analysis by gas chromatography (GC). After complete degradation of CH_3_Cl, vials were ventilated, evacuated and flushed with synthetic air as before, and again CH_3_Cl was added. Microcosms were monitored for CH_3_Cl depletion at regular intervals until an incorporation of 100 μmol carbon per gram sample (soil or degraded leaves) was achieved. Three biological replicated microcosms were then destructively sampled and frozen at − 20 °C for subsequent DNA isolation.

### Gas chromatography

CH_3_Cl was measured in headspace gas volume by injecting 100 μL of a headspace gas sample into a ISQTM Quadrupole GC–MS System using a TRACETM Ultra gas chromatograph (Thermo Fisher Scientific, USA) fitted with a 60 m, 0.32 mm GS-GasPro capillary column (Agilent Technologies, California, USA) with helium as the carrier gas (constant column flow rate, 1.5 mL min^−1^). Headspace gas samples (100 μL) were injected into the column with a temperature ramp from 40 to 200 °C (15 °C increase min^−1^). High-throughput measurements were carried out by the MultiPurpose autosampler MPS (Gerstel Inc., USA). Chromatograms were analyzed with the software Openchrome® (Lablicate GmbH, Germany). CH_3_Cl concentrations were calculated by regression analysis based on a six-point calibration with CH_3_Cl standard ranging from 10 to 150 mM.

### DNA isolation, ultracentrifugation, and high-throughput sequencing

DNA extractions from unincubated samples (T_0_) and samples after incubation with CH_3_Cl were carried out using the FastDNA Spin Kit for Soil (MP Bio Science, Derby, UK). ^13^C-labelled heavy DNA was subsequently separated from unlabeled light ^12^C-DNA using a caesium chloride density gradient ultracentrifugation, as described previously [27]. In brief, a ceasium chloride gradient solution was mixed with the RNA-free DNA from each sample and loaded into an ultra-centrifuge tube. The tube was then placed in a Vti 65.2 vertical rotor (Beckman Coulter, Germany) and centrifuged for 40 h at 177,000x*g* in a LE-70 ultracentrifuge (Backman Coulter, Germany). DNA was then harvested according to established procedures in 12 gradient fractions [27]. Density formation across these 12 fractions (200 μL each) was confirmed by measuring refractive indices using a digital refractometer (Reichert AR2000). DNA was subsequently precipitated from caesium chloride using PEG6000 and glycogen, as described previously [27].

Pre-screening of ‘heavy’ and ‘light’ DNA-SIP fractions of the [^13^C]-CH_3_Cl and [^12^C]-CH_3_Cl incubations and of T_0_ and a “Kitome” (no added sample to detect contaminants from the DNA extraction kit and reagents) sample was carried out using Terminal Restriction Fragment Length Polymorphism (T-RFLP) as described previously [28]. T-RFLP confirmed ‘heavy’ and ‘light’ DNA-SIP fractions, which were subsequently used for 16S rRNA amplicon sequencing on an Illumina Miseq platform at LGC Genomics GmbH, Berlin, Germany. 16S rRNA sequencing of confirmed ‘heavy’ and ‘light’ DNA-SIP fractions can provide insights into the identity of active CH_3_Cl degraders, since these would be enriched in the ^13^C-heavy DNA fractions. Furthermore, using 16S rRNA amplicon sequencing provides an additional confirmation of the ‘heavy’ and ‘light’ DNA-SIP fractions. Amplicon reads were analysed using the QIIME pipeline, and singletons and chimaeras were removed using USEARCH v7 [29] and UCHIME [30]. OTU binning was carried out against the GreenGenes database [31].

### Metagenomics and bioinformatics

Metagenome sequencing was carried out using DNA from [^13^C]-CH_3_Cl-amended microcosms in three biological replicates, together with three replicates of [^12^C]-CH_3_Cl-amended microcosms. Library preparation and sequencing were performed at LGC Genomics GmbH, Berlin, Germany.

Reads of Raw Fastq files were trimmed for the presence of Illumina adapter sequences using Cutadapt version 1.2.1 [32]. The reads were further trimmed by Sickle version 1.200 [33] applying a minimum window quality score of 20. Quality trimmed metagenome reads were then assembled using metaSPAdes v3.11.1 [34] and binned with MyCC version MyCC_2017 [35] using default settings. Estimation of genome completeness and contamination was carried out using the CheckM program [36]. Taxonomic assignment of each bin was carried out by submitting bins to the Rapid Annotation using Subsystem Technology (RAST) annotation pipeline (‘Classic RAST’ pipeline) [37]. Average Nucleotide/Amino Acid Identity (ANI/AAI) [38] between different genomes was estimated using one-way ANI (best hit) and two-way ANI (reciprocal best hit) based on Goris et al. [39]. In order to infer phylogenetic relatedness whole genome phylogenetic trees were constructed using Composition Vector Tree (CVTree) [40]. Furthermore, Bins were screened for 16S RNA-encoding gene sequences using rnammer version 1.2 [41] with option -S bac and -m ssu. To search for presence of functional genes involved in CH_3_Cl degradation within the bins, bins were annotated using Prokka (v1.12) [42] and BlastP [43] searches (cutoff 1e − 30, > 70% identity, manual check of chromosomal region) were carried out against annotated bins (MAGs) using characterized proteins of *cmuA*. Furthermore, ShortBRED (version 0.9.3) [44] and GraftM (version 0.13.0) [45] were used to identify *cmuA* in unassembled metagenomic reads. To estimate the distribution of MAGs in public available datasets, metagenomics reads from various regions (Additional file 1: Table S2) were downloaded from sequence read archive (SRA) using sra-tools 2.8.0. Metagenomic short reads were mapped to Mineral Soil Bin2 using BBmap (v38.79) [46] with an identity threshold of 97% (–idfilter = 0.97). Bedtools intersect (v2.29.2) [47] was used to exclude regions that also displayed high identity alignments of length > 50 bp to the RefSeq database. These ambiguous regions were previously identified by aligning the bin against ncbi’s RefSeq database (downloaded 26.08.2021) using blast + [48] and filtering for pident ≥ 97 (detailed code: https://github.com/SonWende/MAG2metagenome).

### Accession numbers for datasets

SIP 16S rRNA amplicon sequencing and SIP metagenome read data of the Steigerwald soils and degraded leaves have been submitted to the National Center for Biotechnology Information (NCBI) under the BioProject number PRJNA742226.

## Results and discussion

We measured the CH_3_Cl degradation potential and identified the bacterial players involved in CH_3_Cl degradation in deciduous forest soil samples by stable isotope labelling and probing experiments using leaf litter, degraded (senescent, partially decomposed) leaves, organic and mineral soil. These samples were incubated in triplicates with either [^13^C]-CH_3_Cl or [^12^C]-CH_3_Cl and CH_3_Cl concentrations were monitored over 650 h.

### CH_3_Cl degradation potential is highest in top layers of the forest floor

Rapid and steady consumption of CH_3_Cl was observed in all the tested samples of leaf litter, degraded leaves, organic and mineral soils. Degraded leaves and organic soil displayed the highest CH_3_Cl dissipation rates around 1.47 and 1.40 mM CH_3_Cl g^−1^ DW h^−1^ in [^13^C]-CH_3_Cl supplemented condition, respectively (Additional file 2: Figure S1A). CH_3_Cl dissipation rates in [^12^C]-CH_3_Cl incubated samples reached 2.05 and 2.02 mM CH_3_Cl g^−1^ DW h^−1^ in degraded leaves and leaf litter, respectively (Additional file 2: Figure S1B). On the other hand, the lowest CH_3_Cl dissipation rates were observed with the mineral soil (Additional file 2: Figure S1A, B). Furthermore, control incubations with potassium cyanide did not lead to any CH_3_Cl degradation, confirming that the process is biotic (Additional file 2: Figure S2). Thus, the top layers of the forest ground (leaf litter, degraded leaves, and organic horizon) are the most active layers for CH_3_Cl degradation in the tested conditions, as previously found in a study of the same deciduous forest sampling site by Chaignaud et al. 2018 [23].

### Identification of 2 bacterial genome bins exclusively found in [^13^C]-CH_3_Cl treated microcosms

In order to gain insights into the active CH_3_Cl-degrading community in the forest soil samples, DNA-SIP microcosms using [^13^C]-CH_3_Cl or [^12^C]-CH_3_Cl were combined with high-throughput sequencing of the 16S RNA ‘heavy’ and ‘light’ fractions and of the metagenomic DNA of [^13^C]-CH_3_Cl and [^12^C]-CH_3_Cl fractions. 16S rRNA amplicon sequencing showed e.g., an enrichment of *Actinobacteria* in the [^13^C]-CH_3_Cl ‘heavy’ fractions compared to [^13^C]-CH_3_Cl ‘light’ fraction (Additional file 2: Figure S4).

**Fig. 1:**
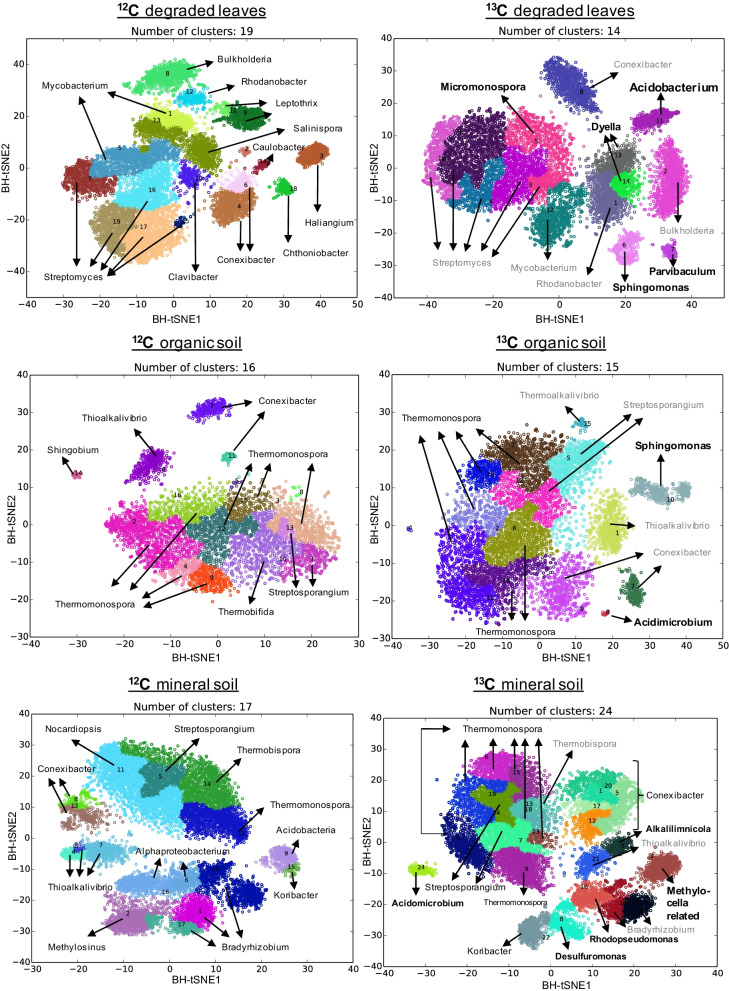
Visual comparison of binned ‘heavy’ control [^12^C]-CH_3_Cl and labelled [^13^C]-CH_3_Cl incubated metagenomes from degraded leaves, organic soil and mineral soil. Microbial names that are written in grey in the [^13^C]-CH_3_Cl‘heavy’ incubation sample are present in ^12^C- and ^13^C-‘heavy’ fractions and are therefore not involved in CH_3_Cl degradation. Microbial names that are written in black are enriched in the ^13^C-‘heavy’ fraction compared to the ^12^C-‘heavy’ fraction. Names that are written in bold are microorganisms that are present exclusively in ^13^C-‘heavy’ fractions and therefore most likely involved in the degradation of CH_3_Cl and were used for further analysis

In order to get a deeper insight into the CH_3_Cl-degrading microorganisms and their metabolic capabilities metagenomic sequencing was carried out using the combined biological replicates of each 12C-and 13C-labelled ‘heavy’ SIP DNA from organic soil, mineral soil and degraded leaf samples. Metagenome reads (~ 33 million read pairs per sample) were assembled and assigned into individual bins (Fig. 1). This resulted in the assignment of a total of 105 bins, comprising of 14–24 bins from each ‘heavy’ fraction ([^13^C]-CH_3_Cl and [^12^C]-CH_3_Cl ‘heavy’ fractions for degraded leaf samples and organic and mineral soil samples). Two taxa of ^13^C-labelled bacterial genome bins did not occur in the ^12^C control treatments and occurred exclusively in the [^13^C]-CH_3_Cl treatment: i.e. ‘Mineral Soil Bin 2’ and ‘Degraded leaves bin 11’ (Fig. 1), which indicates that these are involved in the cycling of CH_3_Cl. We reconstructed genome fragments (so called metagenome assembled genomes, MAGs) of the [^13^C]-CH_3_Cl ‘heavy’ samples, and chose 7 MAGs with the highest quality (> 70% completeness and < 10% contamination) for further analyses (Additional file 2: Table S1). Among those, ‘Mineral Soil Bin 2’ and ‘Degraded leaves bin 11’ harboured no 16S RNA-encoding genes. Taxa assignment was proposed according to the closest representative found in the RAST database (Additional file 2: Table S1). The first represented a member of the Alphaproteobacteria (retrieved from mineral soil) and the second represented a member of *Acidobacteria* (retrieved from the organic layer and degraded leaves). We reconstructed—based on annotations—relevant metabolic pathways of these two MAGs and refined their phylogenetic affiliation.

### Recovery of a population genome of putatively novel family of Alphaproteobacteria retrieved from metagenomes of ^13^C-labelled DNA

**Fig. 2:**
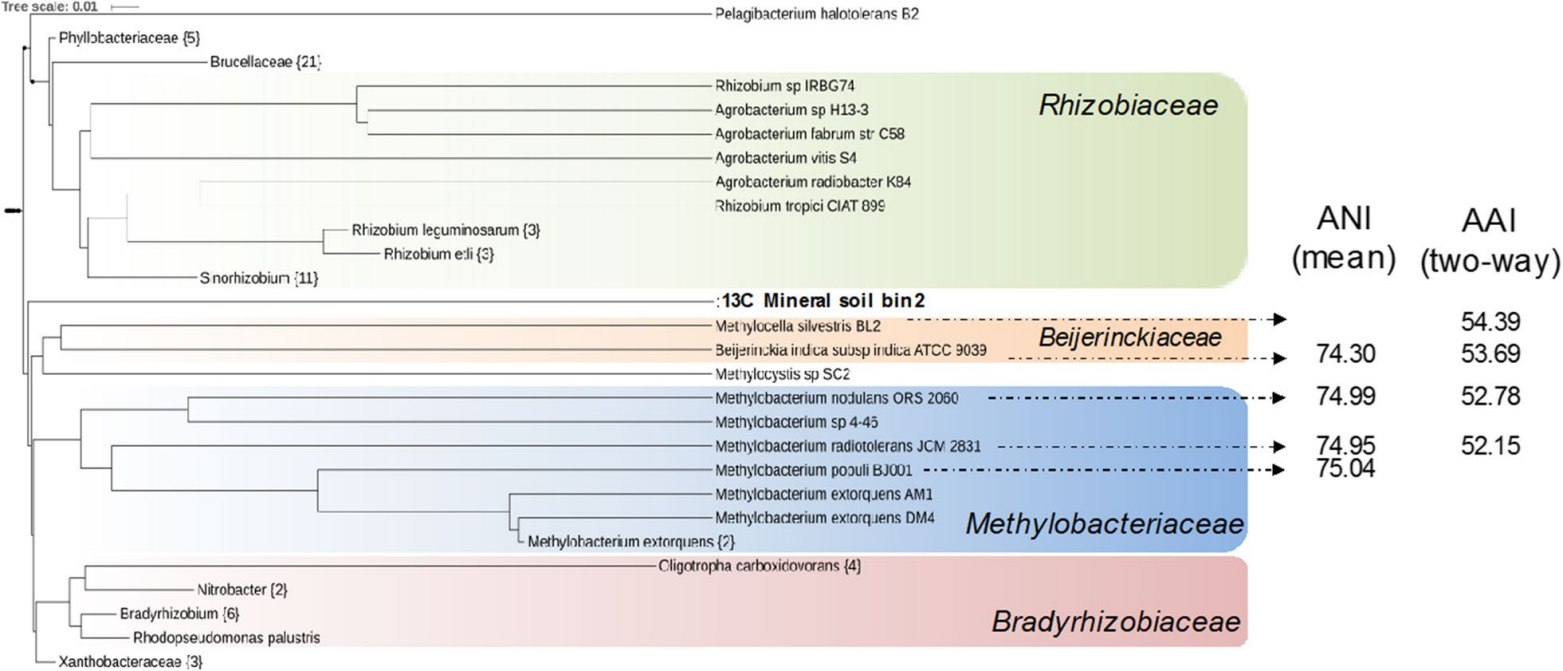
Phylogenetic analysis of whole genome sequences, average nucleotide identity (ANI) and average amino acid identity (AAI) analyses [34] suggest that the MAG “Mineral soil bin 2” is a novel *Alphaproteobacterium*

The MAG exclusively found in the [^13^C]-CH_3_Cl-labelled mineral soil metagenome was related to genomes of *Methylocella* (Mineral Soil Bin2) (Fig. 1). This MAG had a completeness of about 75% and a contamination of about 4% (Additional file 2: Table S1). A phylogenetic tree based on whole genome sequences suggested that this MAG most likely represents a new family closely related to the families *Beijerinckiaceae* and *Methylobacteriaceae* within the class of Alphaproteobacteria (Fig. 2). We performed comparative genome analyses of average nucleotide identity (ANI) and average amino acid identity (AAI) against closely related genomes from the families *Beijerinckiaceae* and *Methylobacteriaceae*, and the data placed this MAG in a novel clade (Fig. 2).

**Fig. 3:**
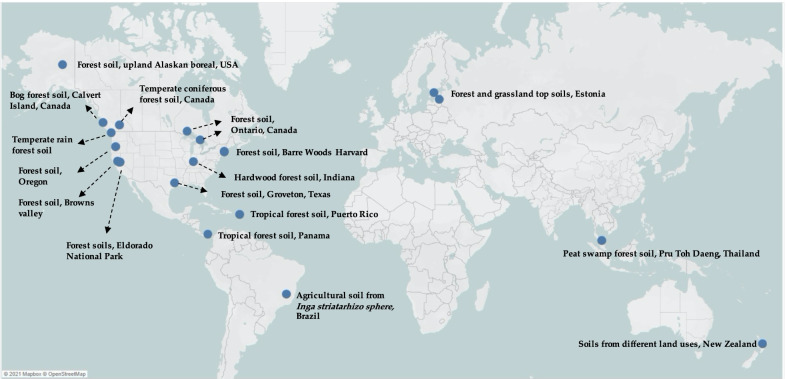
Global distribution of ‘Mineral soil bin 2’ MAG in forest, grassland and agricultural soil. The map was created with Tableau 2021.3

The MAG showed 54.4% AAI to the closely related bacterium *Methylocella silvestris* and between 52 and 54% AAI to genomes of the family *Methylobacteriaceae* (Fig. 2). Therefore, phylogenetic analyses of the whole genome and ANI analysis both strongly suggest that this unclassified group of CH_3_Cl-enriched bacteria forms a novel family within the class of Alphaproteobacteria. Similarly, in the previous study by Chaignaud et al. [23] on the same deciduous forest soil, microorganisms related to *Beijerinckiaceae* were also enriched in CH_3_Cl-amended stable isotope experiments and only identified on the basis of a partial 16S rRNA sequence so that its phylogenetic positioning remained approximate. To assess the breath of distribution of ‘Mineral Soil Bin 2’ in the environment, genome mapping was performed by recruiting metagenomic reads from environmental metagenomes using its ~ 75% complete genome. Total number of reads that are mapped to ‘Mineral Soil Bin 2’ can be highly variable (Additional file 1: Table S2) but detected at high abundance in forest soil samples across the globe (Fig. 3). Moreover, reads mapped to the ‘Mineral Soil Bin 2’ were also detected in soil with different land uses (Fig. 3). Highly related OTUs to the closely related *Beijerinckia* detected by Chaignaud et al. are present in the same forest soil samples across the globe (Additional file 1: Table S2).

**Fig. 4:**
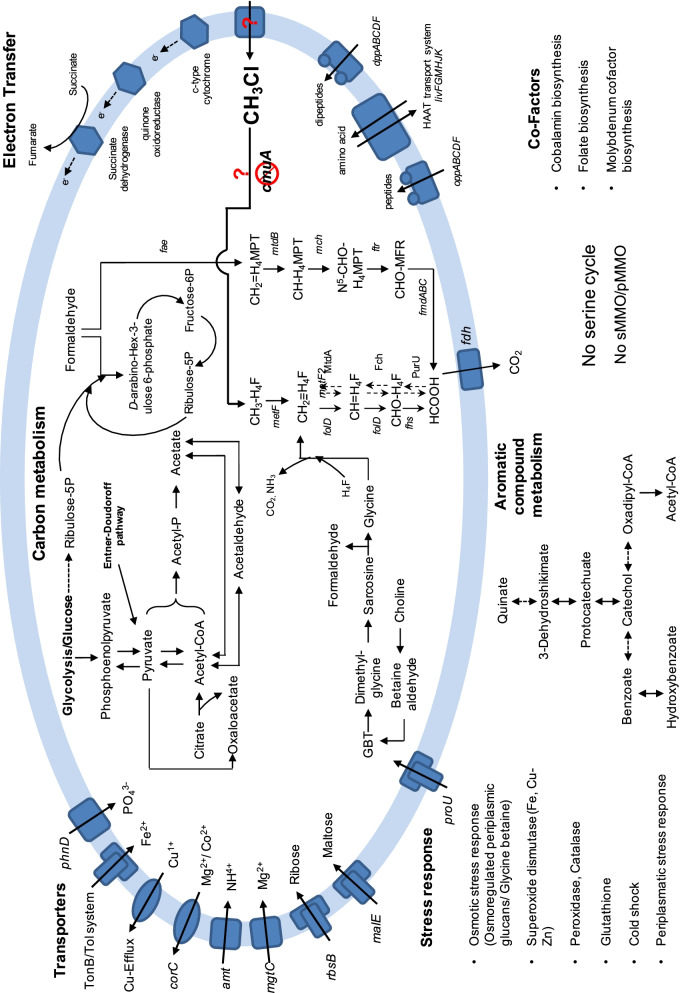
Metabolic reconstruction of [^13^C]-CH_3_Cl labelled metagenome assembled genome “Mineral soil bin 2”. *GBT* glycine betain, *H*_*4*_*F* tetrahydrofolate, *H*_*4*_*MPT* tetrahydromethanopterin, *CHO-MFR* M-formylmethanofuran, *sMMO* soluble methane monooxygenase, *pMMO* particulate methane monooxygenase

Reconstruction of the metabolic pathways revealed that the ‘Mineral soil bin 2’ MAG contained two known pathways to oxidize aerobically methyl-groups to carbon dioxide (Fig. 4)—the tetrahydrofolate (H_4_F) and the tetrahydromethaneopterine (H_4_MPT)-dependent formaldehyde oxidation pathways. However, we could not identify any genes that may code for a dehalogenating enzyme, such as genes *cmuAB* (assessed by BLAST searches and via ShortBRED and GraftM—data not shown). This may be explained by the *cmu* gene plasmid location, as found in *M. extorquens* CM4 [49], which might have thereby escaped metagenome assembly and binning within this MAG. Also, since Chaignaud et al. could not detect *cmuA* from *Beijerinckiaceae* via PCR amplification, the authors suggested that it uses another CH_3_Cl degradation pathway [23]. Despite not having characterized a dehalogenating enzyme, the same predominantly ^13^C-labelled taxon may have been labelled in this study and in the previous SIP study by Chaignaud et al. [23] as both studies were conducted with the same forest soil sampling site. Thus, we conclude that this ‘Mineral soil bin 2’ MAG is most likely responsible for the degradation of the methyl-group CH_3_Cl in these deciduous forest mineral soil samples. Nonetheless, the CH_3_Cl dehalogenating enzyme has yet to be characterized in this taxon.

The ~ 75% complete genome sequences retrieved from the [^13^C]-CH_3_Cl DNA SIP-derived MAG provided the opportunity to explore the metabolic potential in this novel bacterium. Figure 4 summarizes its reconstructed metabolic pathways. Complete gene sets for gluconeogenesis, glycolysis and the Entner-Doudoroff pathway were found. Acetyl-phosphate, which is channelled to the central carbon metabolism through acetyl-CoA is converted to generate ATP, and the gene encoding an acetate kinase is also present in the genome. A gene coding for a succinate dehydrogenase is also found. This MAG contains the genes for two known pathways for oxidation of methyl-groups—the H_4_F and the H_4_MPT-dependent pathways, which result in the formation of formate and subsequent carbon dioxide. Furthermore, genes encoding for the Ribulose Monophosphate (RuMP) pathway are encoded in this MAG. Genes for the serine cycle or a methane monooxygenase (sMMO/pMMO) were not detected. On the other hand, several alcohol dehydrogenases (EC 1.1.1.1) were found in the MAG. In situ and microcosm studies on forest soils pointed towards soil methylotrophs utilizing CH_3_Cl at low concentration in ppt range that may lead to a limitation of energy conservation needed for bacterial growth [22]. In soils, CH_3_Cl amounts for methylotrophic consumption are much lower than methanol, and key CH_3_Cl degraders use alternative carbon sources such as methanol to subsidize the energy requirements [23].

Many *Methylobacteriacea* are motile and possess a single polar, subpolar or lateral flagellum [50]. The ‘Mineral soil bin 2’ MAG does not possess genes encoding for flagellar proteins and is therefore distinct from many members of the closely-related *Methylobacteriaceae* (Figs. 2, 4). In addition, *Methylobacteriaceae* normally possess the serine cycle for formaldehyde assimilation [51], which is also absent in ‘Mineral soil bin 2’ MAG (Fig. 4) and hence distinct from these members. Some members of the family *Beijerinckiaceae*, such as *Beijerinckia* and *Methylocella* are diazotrophs [52] with the ability to reduce nitrogen (N_2_) to ammonia (NH_3_) or ammonium (NH_4_^+^). The closely related ‘Mineral soil bin 2’ MAG does not possess genes encoding for nitrogen fixation and is therefore distinct from members of *Beijerinckia* and *Methylocella* (Figs. 2, 4). These observations strengthen our conclusion that this on CH_3_Cl enriched MAG represents a novel family within the class of Alphaproteobacteria.

### A second ^13^C-labelled MAG suggested involvement of Acidobacteria in C fluxes from CH_3_Cl

The second MAG ‘Senescent Leaves bin 11’ was highly similar to genomes of *Granulicella mallensis* (subdivision *Acidobacteria*), of which members are Gram-negative aerobe rods that have been isolated from soils [53]. The high relatedness of ANI > 87% suggests that it represents a strain of this species (Additional file 2: Figure S5). Here, a H_4_F-dependent pathway for formaldehyde oxidation was present (Additional file 2: Figure S6). This pathway is well known to function for formaldehyde detoxification. Whether the MAG ‘Senescent Leaves bin 11’ was labelled by ^13^C-carbon dioxide or even formaldehyde cross-feeding or directly through utilization of CH_3_Cl cannot be excluded. Nonetheless, MAG ‘Senescent Leaves bin 11’ was ^13^C-labelled.

## Conclusions

Our results suggest that a new key family of aerobic Alphaproteobacteria*,* ‘Mineral soil bin 2’ MAG, is responsible for the degradation of CH_3_Cl. However, this organism has not been cultivated and the mechanisms of CH_3_Cl dehalogenation have not been identified. We did not detect *cmu* genes in the MAG corresponding to this taxon. Furthermore, no *cmuA* genes were detected in the unassembled metagenomic reads, which strongly suggests an alternative *cmu*-independent mechanism or co-metabolic conversion through other soil organisms. In order to identify pathways that were responsible for the degradation of CH_3_Cl in this and in other studies, in which *cmuA* was absent, a comparative transcriptomic and proteomic approach in combination with SIP might be useful.

Combining DNA-SIP with metagenomics and assembly allowed the retrieval of a genome of a putatively novel family of Alphaproteobacteria involved in CH_3_Cl degradation in a deciduous forest soil. The results presented in this work demonstrated the power of multi-methodological approaches combining stable isotope labelling and omics to uncover metabolic functions in as-yet uncultivated novel environmental microbes.

## Supplementary Information

**Additional file 1**. **Table S2**: Metagenomes used for read-recruitment against ‘Mineral soil bin 2’ MAG and the closely related OTU from Chaignaud et al. 2018.

**Additional file 2**. **Figure S1**: CH_3_Cl degradation in deciduous forest soil samples during SIP incubations. **Figure S2**: CH_3_Cl degradation in control deciduous forest soil samples during SIP incubations. **Figure S3**: Relative distribution of terminal restriction fragments detected by bacterial T-RFLP of fractionated DNA from [^13^C]-CH_3_Cl and [^12^C]-CH_3_Cl SIP incubations of mineral and organic soil samples and degraded leave samples from Steigerwald after incorporation of 100 μmol C g^-1^ sample, **Figure S4**: Relative distribution and phylogenetic affiliation of OTUs detected by bacterial 16S rRNA high-throughput amplicon sequencing of samples before SIP experiments (T0) and fractionated DNA from [^13^C]-CH_3_Cl and [^12^C]-CH_3_Cl SIP incubations of mineral and organic soil samples and degraded leave samples from Steigerwald after incorporation of 100 μmol C g^−1^ sample. **Figure S5**: Average nucleotide identity (ANI) analysis of *“*Degraded leaves bin 11*”* and the closely related *Granulicella mallensis* and AAI analysis between those two species. **Figure S6:** Metabolic reconstruction of [^13^C]-CH_3_Cl labelled metagenome assembled genome “Degraded leaves bin 11” (closely related to *Granulicella mallensis*). **Table S1**: Summary of metagenome-assembled genomes (MAGs) from the CH_3_Cl-stable isotope probing enrichment.

## Data Availability

Read data of the Steigerwald soil and degraded leave SIP metagenomes have been submitted to the National Center for Biotechnology Information (NCBI) under the BioProject Number PRJNA742226.
