# Supplementary material for: A putatively new family of alphaproteobacterial chloromethane degraders from a deciduous forest soil revealed by stable isotope probing and metagenomics
Source: Environ Microbiome. 2022 May 8;17:24. doi: 10.1186/s40793-022-00416-2 (PMC9080209; doi:10.1186/s40793-022-00416-2)
Supplement: Supplementary file 2 — Additional file 2. Figure S1: CH3Cl degradation in deciduous forest soil samples during SIP incubations. Figure S2: CH3Cl degradation in control deciduous forest soil samples during SIP incubations. Figure S3: Relative distribution of terminal restriction fragments detected by bacterial T-RFLP of fractionated DNA from [13C]-CH3Cl and [12C]-CH3Cl SIP incubations of mineral and organic soil samples and degraded leave samples from Steigerwald after incorporation of 100 μmol C g-1 sample, Figure S4: Relative distribution and phylogenetic affiliation of OTUs detected by bacterial 16S rRNA high-throughput amplicon sequencing of samples before SIP experiments (T0) and fractionated DNA from [13C]-CH3Cl and [12C]-CH3Cl SIP incubations of mineral and organic soil samples and degraded leave samples from Steigerwald after incorporation of 100 μmol C g−1 sample. Figure S5: Average nucleotide identity (ANI) analysis of “Degraded leaves bin 11” and the closely related Granulicella mallensis and AAI analysis between those two species. Figure S6: Metabolic reconstruction of [13C]-CH3Cl labelled metagenome assembled genome “Degraded leaves bin 11” (closely related to Granulicella mallensis). Table S1: Summary of metagenome-assembled genomes (MAGs) from the CH3Cl-stable isotope probing enrichment. [file 40793_2022_416_MOESM2_ESM.pptx]

## Slide 1
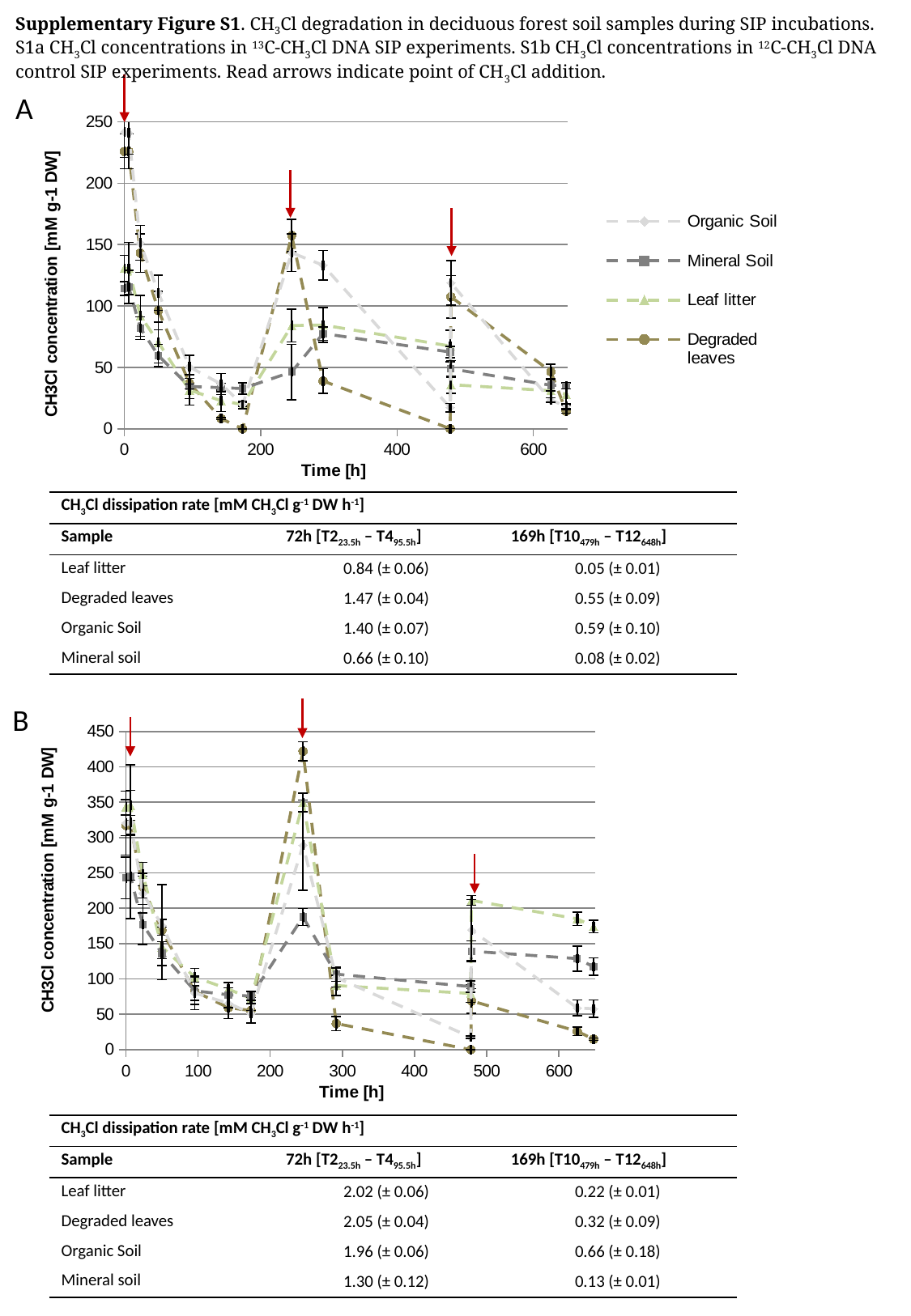

Supplementary Figure S1. CH3Cl degradation in deciduous forest soil samples during SIP incubations. S1a CH3Cl concentrations in 13C-CH3Cl DNA SIP experiments. S1b CH3Cl concentrations in 12C-CH3Cl DNA control SIP experiments. Read arrows indicate point of CH3Cl addition.
### Chart
| Category | Organic Soil | Mineral Soil | Leaf litter | Degraded leaves |
|---|---|---|---|---|A
| CH3Cl dissipation rate [mM CH3Cl g-1 DW h-1] | | |
| --- | --- | --- |
| Sample | 72h [T223.5h – T495.5h] | 169h [T10479h – T12648h] |
| Leaf litter | 0.84 (± 0.06) | 0.05 (± 0.01) |
| Degraded leaves | 1.47 (± 0.04) | 0.55 (± 0.09) |
| Organic Soil | 1.40 (± 0.07) | 0.59 (± 0.10) |
| Mineral soil | 0.66 (± 0.10) | 0.08 (± 0.02) |
B
### Chart
| Category | Organic Soil | Mineral Soil | Leaf litter | Degraded leaves |
|---|---|---|---|---|| CH3Cl dissipation rate [mM CH3Cl g-1 DW h-1] | | |
| --- | --- | --- |
| Sample | 72h [T223.5h – T495.5h] | 169h [T10479h – T12648h] |
| Leaf litter | 2.02 (± 0.06) | 0.22 (± 0.01) |
| Degraded leaves | 2.05 (± 0.04) | 0.32 (± 0.09) |
| Organic Soil | 1.96 (± 0.06) | 0.66 (± 0.18) |
| Mineral soil | 1.30 (± 0.12) | 0.13 (± 0.01) |

## Slide 2
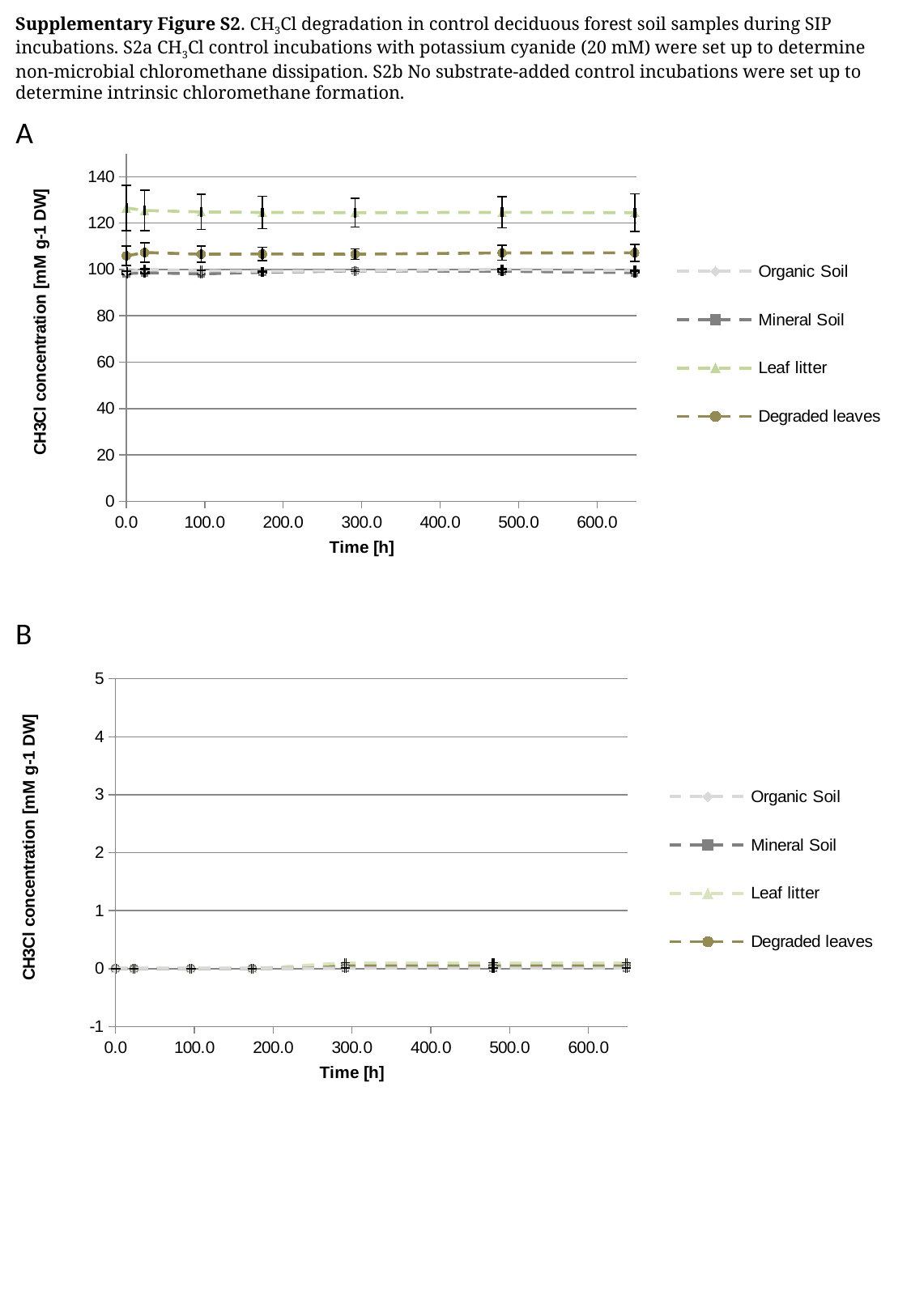

Supplementary Figure S2. CH3Cl degradation in control deciduous forest soil samples during SIP incubations. S2a CH3Cl control incubations with potassium cyanide (20 mM) were set up to determine non-microbial chloromethane dissipation. S2b No substrate-added control incubations were set up to determine intrinsic chloromethane formation.
A
### Chart
| Category | Organic Soil | Mineral Soil | Leaf litter | Degraded leaves |
|---|---|---|---|---|B
### Chart
| Category | Organic Soil | Mineral Soil | Leaf litter | Degraded leaves |
|---|---|---|---|---|

## Slide 3
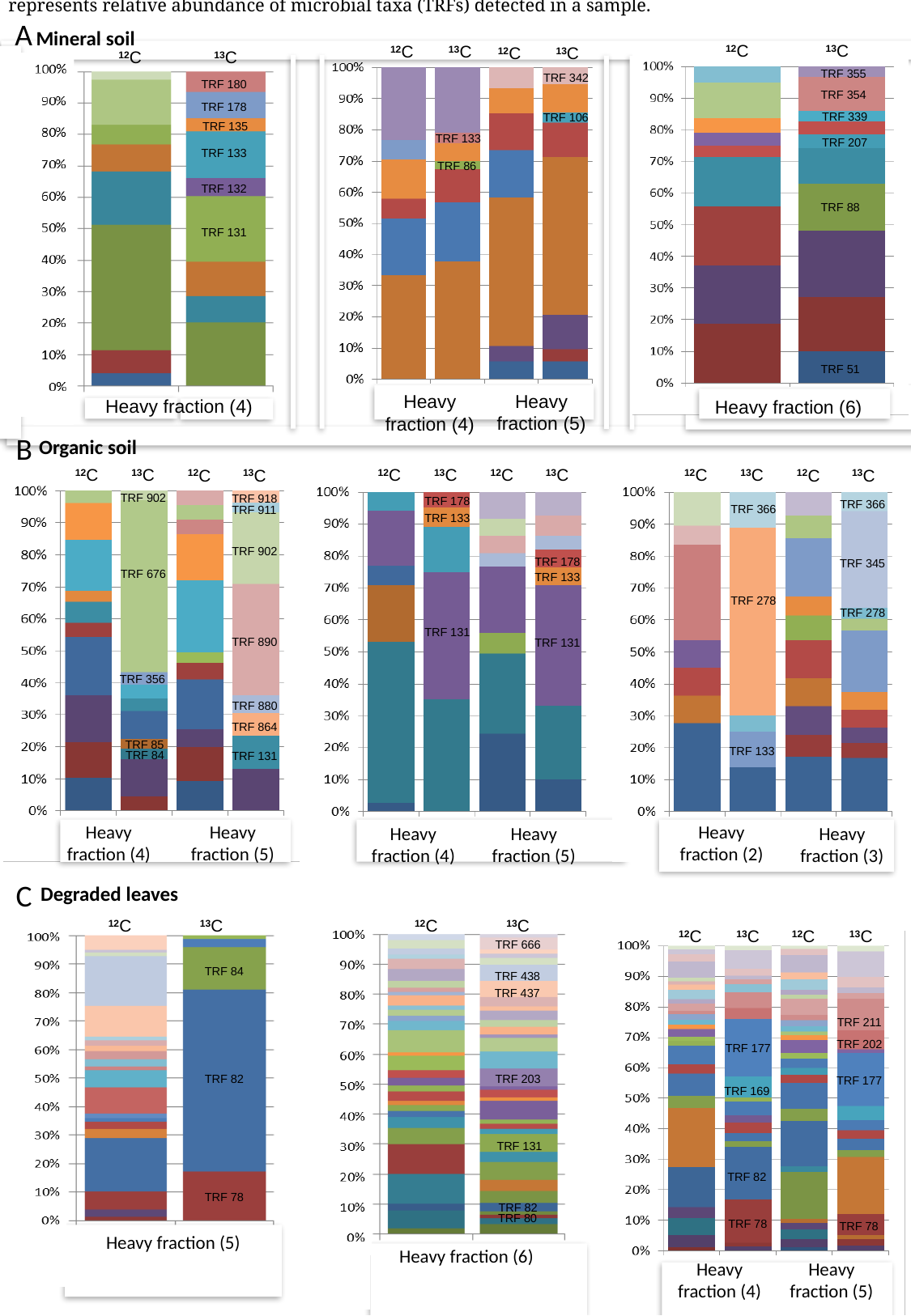

Supplementary Figure S3: Relative distribution of TRFs detected by bacterial T-RFLP of fractionated DNA from [13C]-CH3Cl and [12C]-CH3Cl SIP incubations of mineral and organic soil samples and degraded leave samples from Steigerwald after incorporation of 100 μmol C g-1 sample. Each column represents relative abundance of microbial taxa (TRFs) detected in a sample.
A
Mineral soil
13C
12C
13C
12C
Heavy fraction (4)
13C
12C
12C
13C
Heavy fraction (6)
TRF 342
TRF 180
TRF 178
TRF 135
TRF 133
TRF 133
TRF 86
TRF 132
TRF 131
TRF 355
TRF 354
TRF 339
TRF 106
TRF 207
TRF 88
TRF 51
Heavy fraction (5)
Heavy fraction (4)
B
Organic soil
13C
12C
12C
13C
Heavy fraction (4)
Heavy fraction (5)
TRF 178
TRF 133
TRF 178
TRF 133
TRF 131
TRF 131
13C
12C
12C
13C
Heavy fraction (4)
Heavy fraction (5)
TRF 902
TRF 676
TRF 356
TRF 85
TRF 84
TRF 918
TRF 911
TRF 902
TRF 890
TRF 880
TRF 864
TRF 131
13C
12C
12C
13C
Heavy fraction (2)
Heavy fraction (3)
TRF 366
TRF 366
TRF 345
TRF 278
TRF 278
TRF 133
C
Degraded leaves
12C
13C
TRF 666
TRF 438
TRF 437
TRF 203
TRF 131
TRF 82
TRF 80
Heavy fraction (6)
12C
13C
Heavy fraction (5)
TRF 84
TRF 82
TRF 78
13C
13C
12C
12C
Heavy fraction (4)
Heavy fraction (5)
TRF 211
TRF 202
TRF 177
TRF 177
TRF 169
TRF 82
TRF 78
TRF 78

## Slide 4
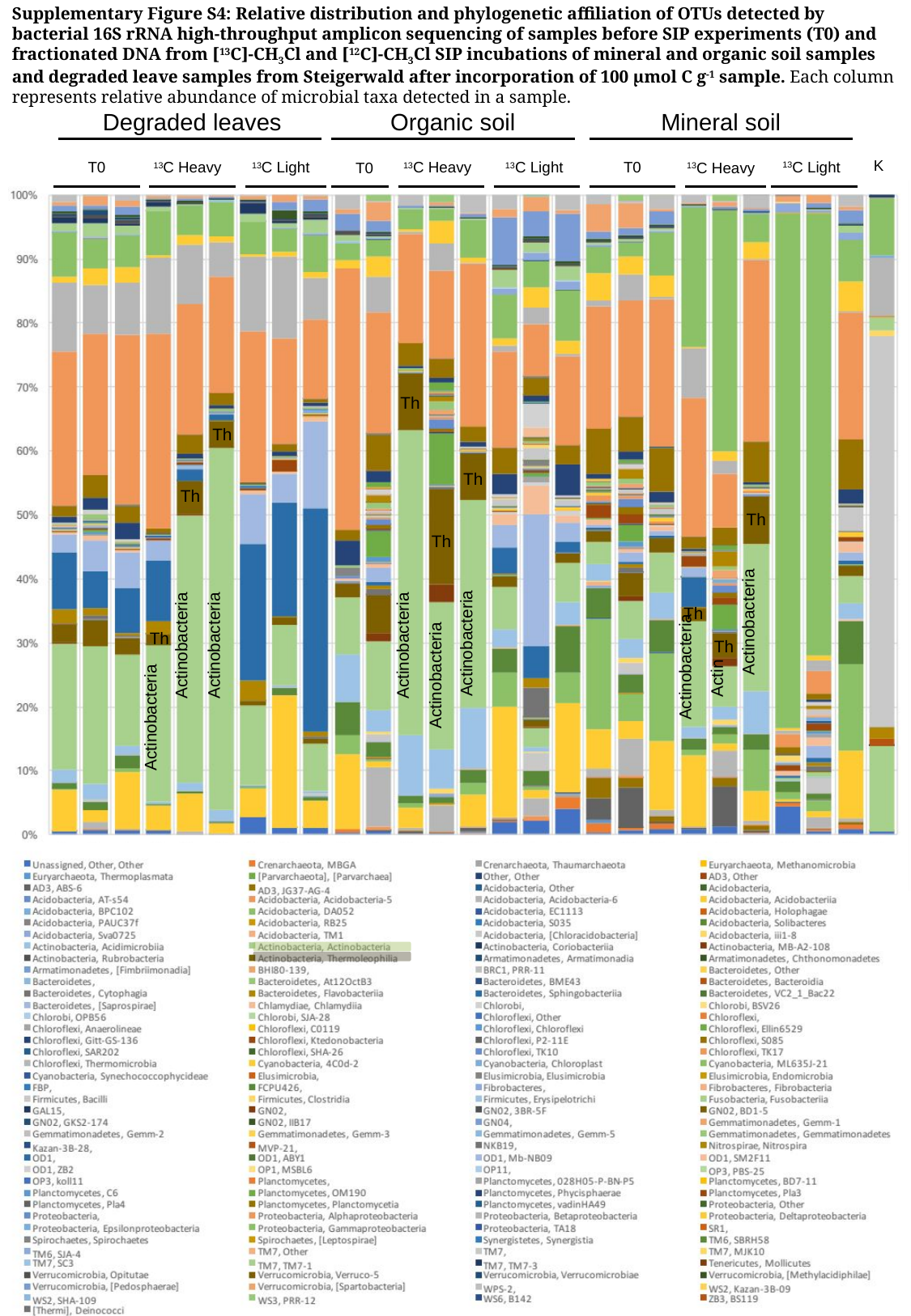

Supplementary Figure S4: Relative distribution and phylogenetic affiliation of OTUs detected by bacterial 16S rRNA high-throughput amplicon sequencing of samples before SIP experiments (T0) and fractionated DNA from [13C]-CH3Cl and [12C]-CH3Cl SIP incubations of mineral and organic soil samples and degraded leave samples from Steigerwald after incorporation of 100 μmol C g-1 sample. Each column represents relative abundance of microbial taxa detected in a sample.
Mineral soil
Organic soil
Degraded leaves
K
T0
13C Light
T0
13C Heavy
13C Light
13C Heavy
13C Light
T0
13C Heavy
Actinobacteria
Actinobacteria
Actinobacteria
Actinobacteria
Actinobacteria
Actinobacteria
Actinobacteria
Actinobacteria
Actin
Th
Th
Th
Th
Th
Th
Th
Th
Th

## Slide 5
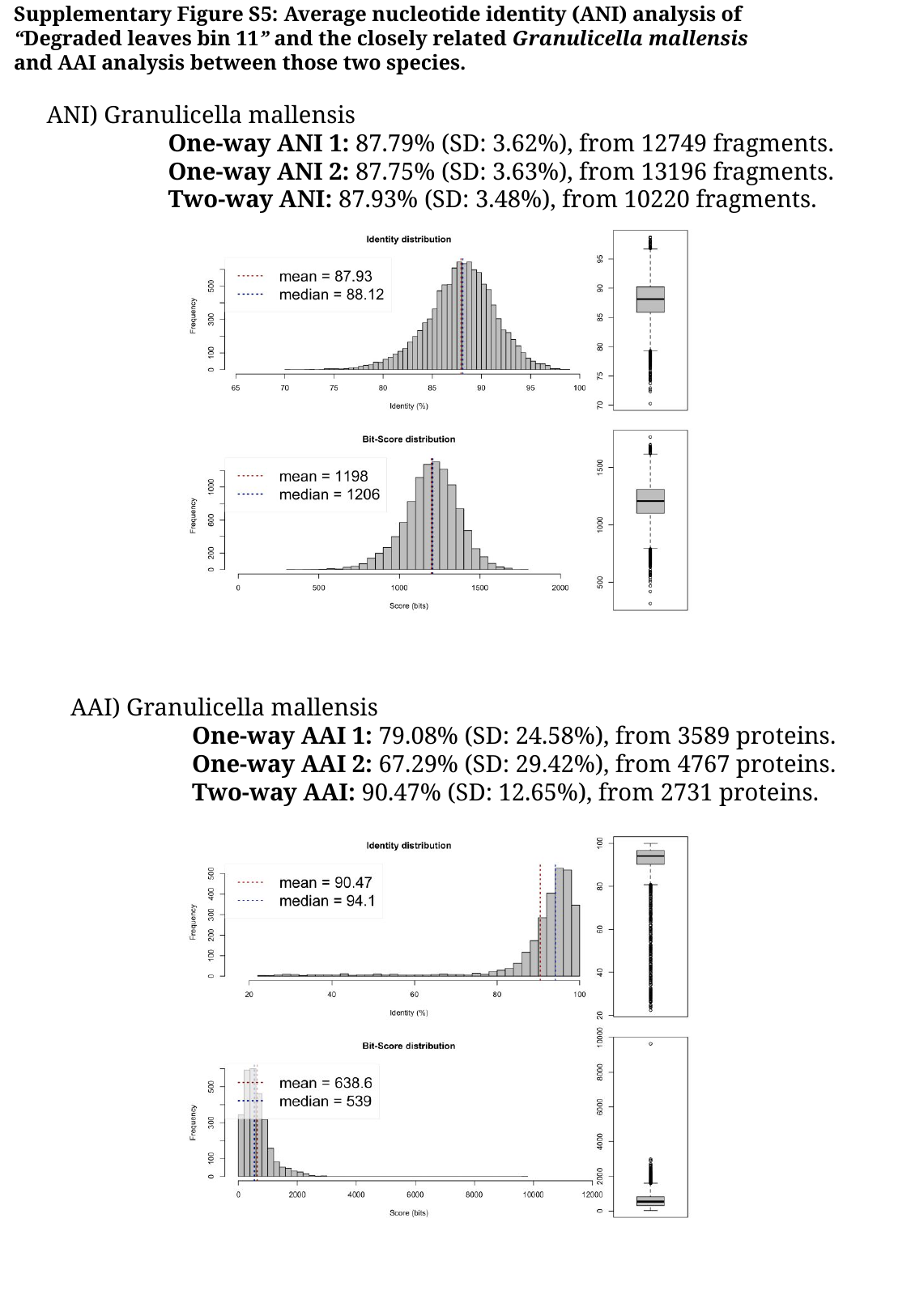

Supplementary Figure S5: Average nucleotide identity (ANI) analysis of “Degraded leaves bin 11” and the closely related Granulicella mallensis and AAI analysis between those two species.
ANI) Granulicella mallensis
	One-way ANI 1: 87.79% (SD: 3.62%), from 12749 fragments.
	One-way ANI 2: 87.75% (SD: 3.63%), from 13196 fragments.
	Two-way ANI: 87.93% (SD: 3.48%), from 10220 fragments.
AAI) Granulicella mallensis
	One-way AAI 1: 79.08% (SD: 24.58%), from 3589 proteins.
	One-way AAI 2: 67.29% (SD: 29.42%), from 4767 proteins.
	Two-way AAI: 90.47% (SD: 12.65%), from 2731 proteins.

## Slide 6
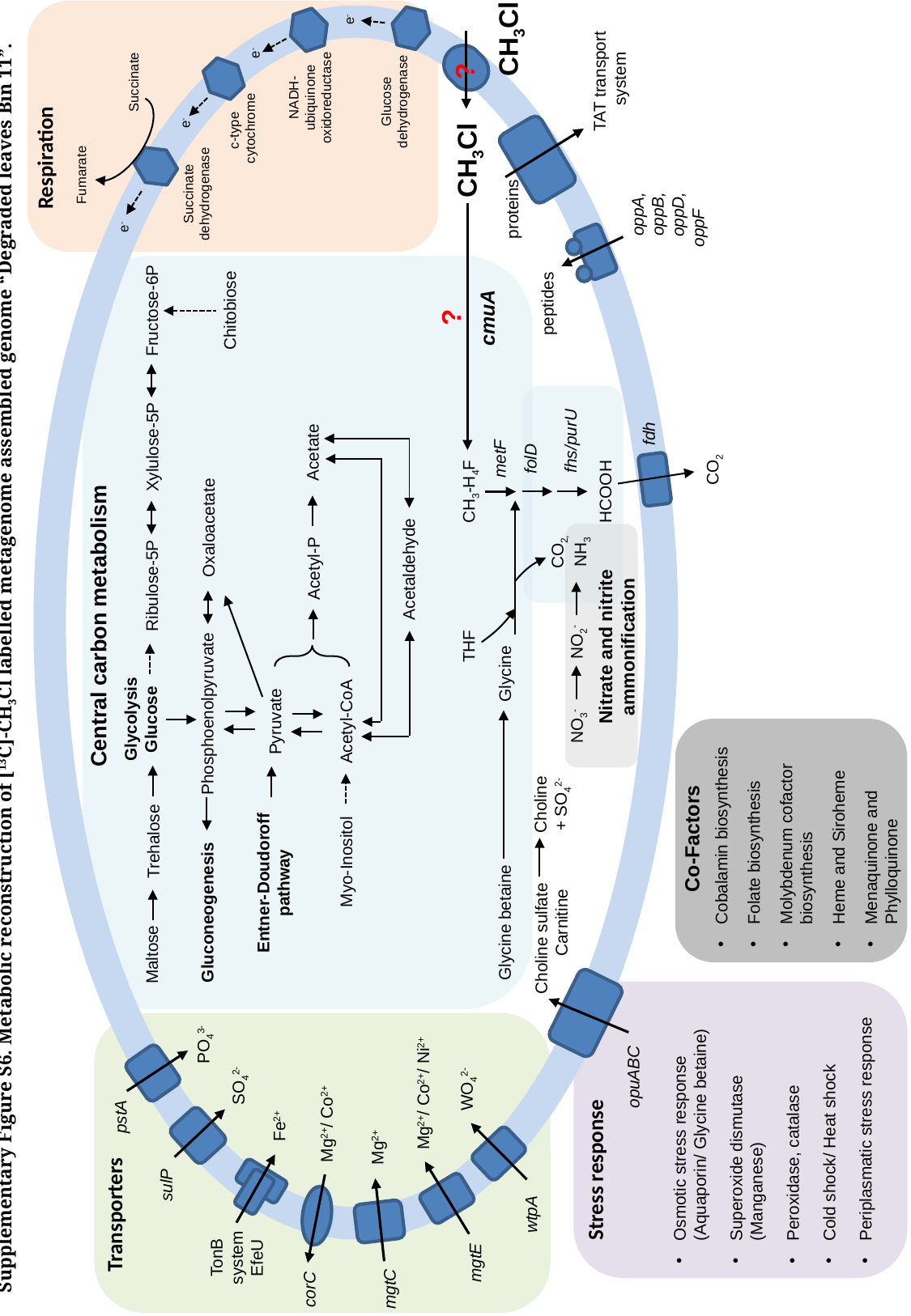

Supplementary Figure S6. Metabolic reconstruction of [13C]-CH3Cl labelled metagenome assembled genome “Degraded leaves Bin 11”.
Respiration
Transporters
TonB system
EfeU
Fe2+
corC
Mg2+/ Co2+
Mg2+
mgtC
Mg2+/ Co2+/ Ni2+
mgtE
WO42-
wtpA
Glycine betaine
Choline sulfate
Carnitine
opuABC
proteins
TAT transport
system
peptides
oppA, oppB, oppD, oppF
Stress response
Osmotic stress response (Aquaporin/ Glycine betaine)
Superoxide dismutase (Manganese)
Peroxidase, catalase
Cold shock/ Heat shock
Periplasmatic stress response
Glycolysis
Glucose
Gluconeogenesis
Phosphoenolpyruvate
Pyruvate
Acetate
Acetyl-P
Acetyl-CoA
Acetaldehyde
Entner-Doudoroff pathway
Ribulose-5P
Fructose-6P
Xylulose-5P
Oxaloacetate
Chitobiose
CH3-H4F
metF
folD
fhs/purU
THF
Glycine
HCOOH
CO2
CO2, NH3
fdh
Maltose
Trehalose
Myo-Inositol
sulP
SO42-
Choline
+ SO42-
pstA
PO43-
Fumarate
e-
Succinate
e-
Succinate dehydrogenase
c-type
cytochrome
e-
NADH-ubiquinone oxidoreductase
e-
Glucose dehydrogenase
NO2-
NO3-
Nitrate and nitrite ammonification
Co-Factors
Cobalamin biosynthesis
Folate biosynthesis
Molybdenum cofactor biosynthesis
Heme and Siroheme
Menaquinone and Phylloquinone
Central carbon metabolism
?
?
CH3Cl
cmuA
CH3Cl

## Slide 7
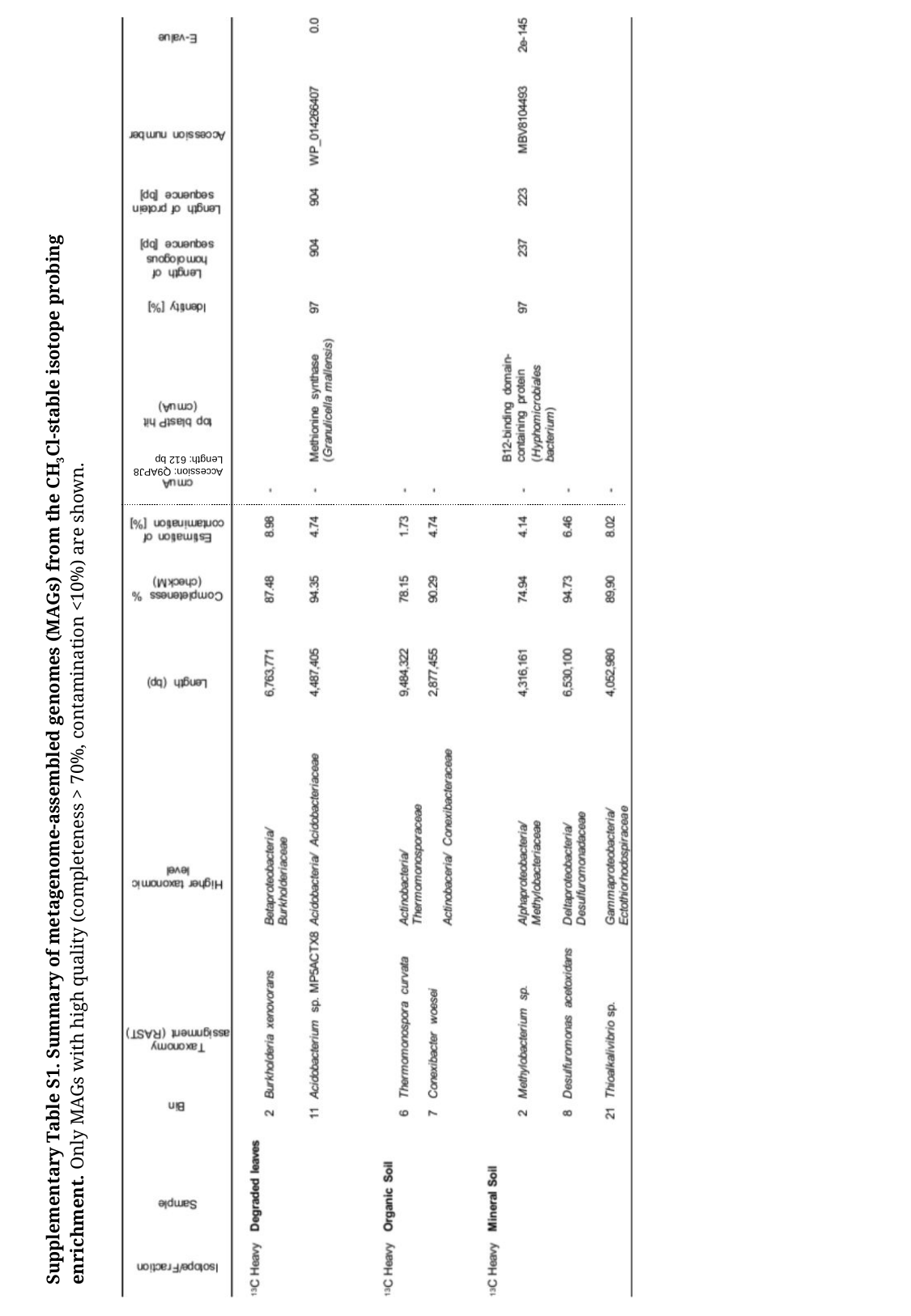

Accession: Q9APJ8
Length: 612 bp
Supplementary Table S1. Summary of metagenome-assembled genomes (MAGs) from the CH3Cl-stable isotope probing enrichment. Only MAGs with high quality (completeness > 70%, contamination <10%) are shown.
